# IMPROVING THE MEASUREMENT OF EXERCISE SELF-EFFICACY TO INFORM INNOVATIVE REHABILITATION TREATMENTS

**DOI:** 10.1093/geroni/igz038.3385

**Published:** 2019-11-08

**Authors:** Patricia Bamonti, Rebekah Harris, Jennifer Moye, Kelly Doherty, Jonathan Bean

**Affiliations:** 1 VA Boston Healthcare, Boston, Massachusetts, United States; 2 NE GRECC, VA Boston Healthcare System, Boston, Massachusetts, United States; 4 New England GRECC, VA Boston Healthcare System, Boston, Massachusetts, United States

## Abstract

Exercise self-efficacy (ESE) is a consistent determinant of exercise behavior but barriers to ESE remain unexplored in medically-complex older adults. This study explored 1) concordance between Physical Therapist’s (PT’s) and patients’ rating of their confidence to exercise; and 2) Whether patient demographic and clinical variables (e.g., depression) were related to confidence ratings. Data were collected as part of a clinical demonstration project, “Live Long Walk Strong,” a PT intervention to prevent mobility decline. Patients (N=35) had a mean age of 78.26 +/- 11.12, were 86% male, 80% White, and manifested an average of 5.63+/-1.96) chronic conditions. They had an average of 3.46+/-5.00, falls in the previous year and a mean M=4.97 (SD=2.96), Geriatric Depression Scale score of 4.97+/-2.96. Patients and PT’s rated exercise confidence (1= not at all to 5 = extremely). Participants’ and PT’s confidence ratings of the patient were highest for exercise in the clinic, followed by at home, and continuing after PT, and were moderately correlated (r = .41-.52, p < .001). PT confidence was associated with younger age (r =.48, p < .001) and lower depression (r = -.35, p = < .05), whereas these variables were not significantly associated with patient confidence. In qualitative analysis patients cite barriers to exercise: (1) physical health such as pain, fatigue, balance/previous falls, weakness; (2) memory and cognition; (3) time; (4) past experiences with PT; (5) poor social support; (6) low self-efficacy. In sum, older adults and PT’s generally agree in their confidence ratings, but barriers to ESE differ.

